# Will another trial CONVINCE nephrologists to adopt high-dose haemodiafiltration over conventional haemodialysis?

**DOI:** 10.1093/ckj/sfad258

**Published:** 2023-10-11

**Authors:** Kaitlin J Mayne, Claudio Ronco

**Affiliations:** Clinical Trial Service Unit and Epidemiological Studies Unit (CTSU), Nuffield Department of Population Health, University of Oxford, Oxford, UK; School of Cardiovascular and Metabolic Health, College of Medical and Veterinary Life Sciences, University of Glasgow, Glasgow, UK; International Renal Research Institute of Vicenza, Department of Nephrology, Dialysis and Transplantation, San Bortolo Hospital, Vicenza, Italy

Haemodialysis (HD) relies on diffusion for solute clearance using either low-flux or high-flux membranes. High-flux dialysers have increased permeability to middle molecular weight solutes. Haemodiafiltration (HDF) employs diffusion and convection in combination thereby allowing even greater clearance of middle and large molecules versus high-flux HD [1]. Both are accepted treatments for kidney failure, but high-flux HD is much more widely used. The enhanced clearance achieved by HDF may improve haemodynamic stability and survival though high-quality randomised evidence is lacking [1].

**Figure 1:**
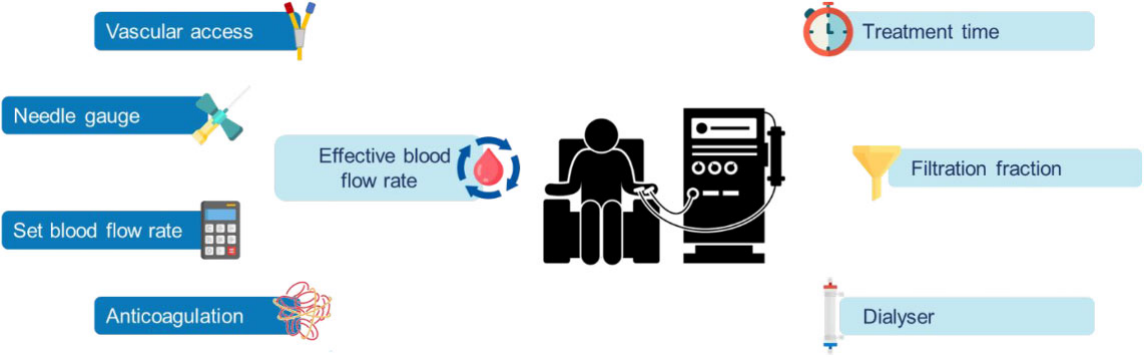
Treatment-related determinants of HDF convection volume.

Previous trials have tested whether high-dose HDF confers survival advantage over high-flux HD but with several limitations [2]. Survival benefits have been linked to higher convection volumes of which there are many determinants [2]; these are largely modifiable treatment-related factors such as vascular access (Fig. 1); however, the observation that higher convection volumes are typically achieved in healthier patients has been cited as a potential confounder in previous trials [2]. The recently reported CONVINCE trial attempted to overcome this limitation by mandating candidacy for convection volumes ≥23 litres in post-dilution mode for enrolment [3, 4]. The trial was set within routine clinical practice and randomised 1360 adults receiving high-flux HD for ≥3 months (median vintage ∼3 years) across 61 European centres to either thrice weekly HDF or continued high-flux HD. Participants were less comorbid than typical haemodialysis populations and >85% had arteriovenous access, likely because suitability for high convection volumes determined inclusion. The 23-litre post-dilution target was reached in 92% of HDF sessions; the overall mean in the intervention group was 25.3 litres. Over a median of 2.5 years, all-cause mortality was reduced by 23% [hazard ratio (HR) 0.77, 95% CI 0.65–0.93, 266 deaths]. The survival advantage of HDF was previously attributed to cardiovascular benefits but CONVINCE found no significant effect on cardiovascular mortality (HR 0.81, 95% CI 0.49–1.33) nor the composite of fatal or nonfatal cardiovascular events (HR 1.07, 95% CI 0.86–1.33); perhaps owing to recruitment of a ‘healthier’ population. Instead the reduction in mortality appeared driven by infection outcomes, largely COVID-19-related, but without a significant difference in hospitalisations (HR 1.11, 95% CI 0.98–1.25). Reduced death from infection is plausible because enhanced middle molecule clearance may reduce inflammation [1]; however, no between-group difference in C-reactive protein was observed nor was clearance of other middle molecules such as beta-2 microglobulin reported [3].

Although promising, the CONVINCE trial results are insufficient to drive widespread adoption of HDF based upon all-cause mortality reduction alone and unclear mechanism of benefit. The EuDial Working Group reached the same conclusion in their eloquent in-depth discussion of the trial's intricacies [5]. Unanswered questions remain: HDF has cost and sustainability implications and understanding which patients are most likely to benefit might inform individualised rather than blanket adoption of HDF. Additional randomised evidence will emerge from the H4RT trial in 2025 [6] (Table 1); pooled analyses may allow greater exploration of subgroup effects. Dialysis treatment remains far from optimal and future technologies might utilise the adsorptive mechanisms employed in continuous kidney replacement therapy in pursuit of optimal solute clearance.

**Table 1:** Summary of the CONVINCE trial and ongoing H4RT trial.

	CONVINCE	H4RT
Status	Results reported June 2023	Completed recruitment Follow-up expected to continue until May 2025
**Population**		
N	1360	1553
Sites	8 European countries	United Kingdom
Eligibility	Adults with kidney failure receiving high-flux HD for ≥3 months ‘Candidate for high-dose HDF’ intervention target	Adults with kidney failure receiving maintenance HD or HDF ≥3 times per week ‘Potential to achieve high-volume HDF’ intervention target
**Intervention target**	‘High-dose’ HDF defined as convection volume ≥23 L^a^	‘High-volume’ HDF aiming for ≥21 L substitution fluid per 1.73 m^2^ body surface area^a^
**Control**	High-flux HD	High-flux HD
**Outcomes**		
Primary outcome	All-cause mortality	Composite of non-cancer death or hospitalisation for cardiovascular or infection-related causes
Additional outcomes	Cause-specific mortality Cardiovascular events Kidney transplantation Hospitalisations (any cause, infection-related) Laboratory parameters Patient-reported outcomes Cost-effectiveness	All-cause, non-cancer mortality Cardiovascular, infection-related hospitalisation and mortality Health-related quality of life Laboratory parameters Cost-effectiveness Environmental impact
**Follow-up**	Median 30 months	Minimum 32 months

a Convection volume (total ultrafiltration volume) equals the sum of substitution volume plus net ultrafiltration volume (the intended removal volume to achieve dry weight). ‘High-dose’ and ‘high-volume’ are considered equivalent terms.
